# A Hybrid Lesion of Lung Cancer and Aspergillosis

**Published:** 2008-04-10

**Authors:** Hiroaki Takeoka, Takeharu Koga, Hirohisa Yano, Jiro Ikeda, Munetsugu Nishimura, Tomoko Kamimura, Hisamichi Aizawa

**Affiliations:** 1 Division of Respirology, Neurology and Rheumatology, Department of Medicine; 2 Kurume Daiichi Social Insurance Hospital, Kurume; 3 Amagi-Asakura Medical Association Hospital, Asakura; 4 Department of Pathology, Kurume University School of Medicine

**Keywords:** lung cancer, aspergillosis, cavitary lung lesion

## Abstract

A 74-year-old man presented with gradual wall thickening of a cystic lung lesion. Serologic tests indicated *Aspergillus* infection, but neither fungal organisms nor evidence of malignant disease were recovered from repeated sputum collections, a bronchoscopic lung biopsy specimen, or bronchial washings. Treatment with antifungal agents did not result in clinical improvement. Surgical resection of the lesion demonstrated both squamous cell carcinoma and aspergillosis. These distinct disorders share common radiologic manifestations that can present a diagnostic challenge, as in the present case.

## Case Presentation

**Figure 1:**
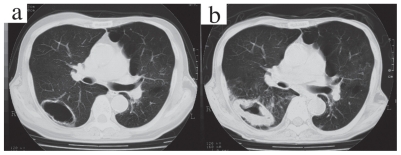
Chest CT in 2003 (**a**) demonstrated a CT thick-walled cystic lesion in the superior segment of the lower lobe of the right lung. A corresponding CT image in 2004 (**b**) demonstrated increased thickening of the wall.

A 74-year-old man sought medical evaluation in May 2004, because of an increasing productive cough. He previously had noted productive cough, but did not become concerned until February 2004, when its frequency and intensity began to increase with no clear triggering episode or factor. He denied any history of hemoptysis, night sweats, pyrexia, or weight loss. Past medical history included pulmonary tuberculosis diagnosed at age 35 and a curative operation in 1995 for oropharyngeal carcinoma requiring reconstruction of the oropharyngeal wall with autologous grafts. Diagnosed with chronic obstructive pulmonary disease at the time of that he quit smoking. He underwent follow-up chest radiography at irregular intervals of 1 to 3 years. In May 2003 the patient was asymptomatic, but a cystic lesion in the right lung showed an increase in wall thickness compared with a previous radiograph obtained in 2000. Computed tomography (CT) of the chest in 2003 (ProSeed; GE Yokogawa Medical Systems, Tokyo) with a conventional protocol without enhancement (10-mm collimation, 10-mm intervals, 120 kVp, 200 mAs, gantry rotation time; 1.0 sec.) showed moderate wall thickening (Fig. 1a). The patient underwent repeated sputum examinations, and then bronchoscopic lung biopsy and bronchial washing targeting the right B6 segment. None of these specimens yielded evidence of cancer or bacterial or fungal pathogens. Further diagnostic procedures were declined. The patient was an office worker with no known history of dust exposure. He had smoked 1.5 packs of cigarettes daily for 42 years until he quit. He denied having undergone any blood transfusions or regular courses of medication.

The patient was 172 cm tall and weighted 55 kg. Vital signs at presentation included body temperature of 36.5 ˚C, blood pressure of 110/78 mmHg, and pulse rate of 72/min with a regular rhythm at resting. Physical examination disclosed a healed surgical scar of the neck and diminished breath sounds in both lungs. Heart sounds included no murmurs. The abdomen was unremarkable. No neurologic findings were present. Finger clubbing was absent. Laboratory data at evaluation in 2004 are summarized in the Table 1. Serologic tests were positive for galactomannan (latex agglutination) and anti-*Aspergillus* antibody (complement fixation).

Chest CT, acquired by the same protocol as in 2003 without enhancement, disclosed diffuse thickening of the cyst wall (Fig. 1b). Infiltrative opacities were seen in the area proximal to the cystic lesion. Results of sputum cytologic examination were negative. Neither endoscopic trans-bronchial lung biopsy nor CT-guided transthoracic needle aspiration could establish a diagnosis. Surgical removal of the lung lesion initially was declined. When the patient was treated with the antifungal agents itraconazole and micafungin for presumed aspergillosis, the lesion continued to enlarge. The patient then agreed to undergo right lower lobectomy after the 2 months delay.

**Figure 2:**
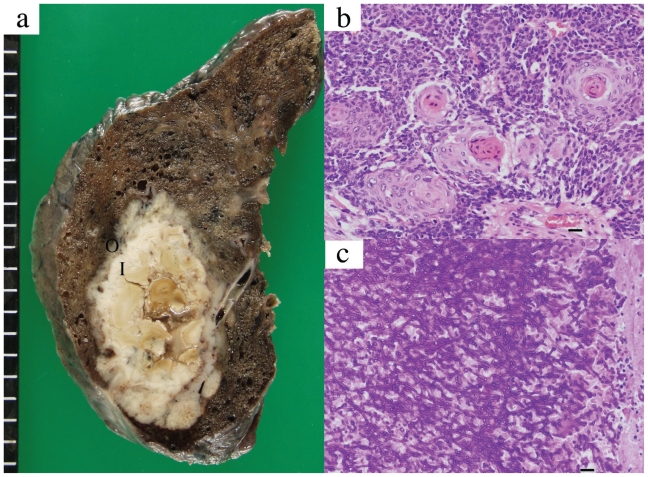
Macroscopic examination of the excised right lower lobe (**a**) showed the mass to have two-layered structure, with outer and inner zones. Microscopically the outer layer, (labeled “O” in (a) showed compact proliferation of atypical cells with “pearl” formation in some areas, characteristic of squamous cell carcinoma (Hematoxylin-Eosin stain, bar = 25 μm) (**b**). The inner layer, (labeled “I” in (a), showed septate, and branching hyphae (Hematoxylin-Eosin stain, bar = 25 μm) (**c**).

Macroscopically the resected lesion was solid, but with an outer and inner component (Fig. 2a). Microscopically, the outer portion (labeled “O” in Fig. 2a) showed dense proliferation of atypical cells with focal “pearl” formation, characteristic of squamous cell carcinoma (Fig. 2b). The inner layer (labelled “I” in Fig. 2a) was composed of septate, branching hyphae (Fig. 2c). Branching at acute angles was suggestive of *Aspergillus* sp. The pathologic tumor stage of the resected lung cancer was T2N0M0. The patient was thus diagnosed as having lung cancer complicated by aspergillosis.

## Discussion

Cystic lung lesions often show increases in wall thickness, either spontaneously (Ryu and Swensen, 2003) or in response to various superimposed conditions such as microbial infections. The cyst in the present case was of uncertain cause, but the smoking history and related pulmonary emphysema suggest that the cyst may have arisen from emphysema. Walls of cystic or cavitary lesions can thicken because of cancer (Tsutsui et al. 1988), mycobacteriosis (Bull et al. 2003), or aspergillosis (Kawamura et al. 2000). *Aspergillus* species often infest cavitary lung lesions, most notably tuberculous cavities, forming spherical saprophytic lesions termed aspergillomas. *Aspergillus* infection of various cystic lesion, including emphysematous bullae, has also been known to manifest lesion wall thickening (Kawamura et al. 2000), as may have occurred in the present case. All three conditions were considered as differential diagnoses in this patient. An assay for circulating galactomannan, a noninvasive aid for diagnosis of *Aspergillus* infection (Maertens et al. 2002), yielded a result exceeding the cutoff value, in combination with a positive complement fixation reaction for anti-*Aspergillus* antibody and an elevated serum β-D-glucan concentration, the result suggested apergillosis as the cause of cyst wall thickening. In a reported case similar to ours, resection of the growing cavitary lung lesion confirmed coexisting lung cancer and aspergillosis; neither was diagnosed preoperatively (McGregor et al. 1989), illustrating that accurate diagnosis can be a challenge. In another case, development of aspergillosis was suggested to have complicated airway obstruction by the tumor (Itano et al. 2005). While either lung cancer or aspergillosis may present a cavitary mass lesion in imaging examinations, a recent study (Park et al. 2007) proposed several radiologic features as useful in discriminating the two diseases. Nonetheless, as in the present patient, their coexistence can be a difficult diagnostic problem.

Occasional patients with bronchogenic carcinoma complicating pre-existing aspergillosis have been reported, usually showing fungal growth within a cavitating carcinoma and necrotic carcinoma cell clusters intermingled with hyphae (McGregor et al. 1989; Torpoco et al. 1976). On the other hand, complication of noncavitating lung cancer by aspergillosis has been described (Yoshitomi et al. 2000). Right lower lobectomy in the present case disclosed a mass composed of twolayers; cancer cells were confined to the outer layer and not intermingled with the fungal hyphae in the inner zone. Whether the central fungal growth represented complete replacement of necrotic tissue tumor or independent development of two different lesions is not certain. However, the two-layered structure suggests that cancer development preceded fungal growth, with progressive wall thickening resulting mainly from lung cancer.

## Figures and Tables

**Table 1:** Laboratory data on presentation.

	Results	Normal values
Haemoglobin g^•^ L^−1^	90	140–180
WBC 10^9^ cells^•^ L^−1^	9.4	4.0–9.0
Platelet count 10^9^ cells^•^ L^−1^	379	130–360
C reactive protein mg^•^ L^−1^	5.77	<0.4
ESR mm^•^ h^−1^	116	<10
ALT U^•^ L^−1^	15	8.0–42
AST U^•^ L^−1^	14	13–33
LDH U^•^ L^−1^	198	119–229
Cholinesterase U^•^ L^−1^	91	107–233
Total protein g^•^ L^−1^	6.88	6.7–8.3
Albumin U^•^ L^−1^	3.09	4.0–5.0
Blood urea nitrogen mg^•^ L^−1^	6.3	8.0–22
Creatinine mg^•^ L^−1^	0.59	0.6–1.1

**Abbreviations:** WBC: white blood cells; ESR: erythrocyte sedimentation rate; ALT: alanine aminotransferase; AST: aspartate aminotransferase; LDH: lactate dehydrogenase.
